# NEOSCOPE: a randomised Phase II study of induction chemotherapy followed by either oxaliplatin/capecitabine or paclitaxel/carboplatin based chemoradiation as pre-operative regimen for resectable oesophageal adenocarcinoma

**DOI:** 10.1186/s12885-015-1062-y

**Published:** 2015-02-12

**Authors:** Somnath Mukherjee, Christopher N Hurt, Sarah Gwynne, Andrew Bateman, Simon Gollins, Ganesh Radhakrishna, Maria Hawkins, Jo Canham, Wyn Lewis, Heike I Grabsch, Ricky A Sharma, Wendy Wade, Rhydian Maggs, Bethan Tranter, Ashley Roberts, David Sebag-Montefiore, Timothy Maughan, Gareth Griffiths, Tom Crosby

**Affiliations:** 1 CRUK MRC Oxford Institute for Radiation Oncology Gray Laboratories, University of Oxford, Old Road Campus Research Building, Oxford, UK; 2 Wales Cancer Trials Unit, School of Medicine, Cardiff University, Cardiff, UK; 3 South West Wales Cancer Centre, Swansea, UK; 4 Cancer Care, University Hospital Southampton, Southampton, UK; 5 Department of Clinical Oncology, North Wales Cancer Treatment Centre, Rhyl, UK; 6 St James’s Institute of Oncology, Leeds, UK; 7 University Hospital of Wales, Cardiff and Vale NHS Trust, Cardiff, UK; 8 Department of Pathology, Maastricht University Medical Centre, Maastricht, The Netherlands; 9 NISCHR CRC South East Wales Research Network, Cardiff, UK; 10 Cardiff NCRI RTTQA group, Department of Medical Physics, Velindre Cancer Centre, Cardiff, UK; 11 Velindre Cancer Centre, Velindre NHS Trust, Cardiff, UK; 12 University of Leeds, Cancer Research UK Leeds Centre, St James’s University Hospital, Leeds, UK; 13 Southampton Clinical Trials Unit, Faculty of Medicine, Southampton University, Southampton General Hospital, Southampton, UK

**Keywords:** Oesophageal, Phase II, Neo-adjuvant, Chemotherapy, Chemoradiotherapy, Radiotherapy, Carboplatin, Paclitaxel, Oxaliplatin, Capecitabine

## Abstract

**Background:**

Both oxaliplatin/capecitabine-based chemoradiation (OXCAP-RT) and carboplatin-paclitaxel based radiation (CarPac-RT) are active regimens in oesophageal adenocarcinoma, but no randomised study has compared their efficacy and toxicity. This randomised phase II “pick a winner” trial will identify the optimum regimen to take forward to a future phase III trial against neo-adjuvant chemotherapy, the current standard in the UK.

**Methods/Design:**

Patients with resectable adenocarcinoma of the oesophagus or Siewert Type 1–2 gastro-oesophageal junction (GOJ), ≥T3 and/or ≥ N1 are eligible for the study. Following two cycles of induction OXCAP chemotherapy (oxaliplatin 130 mg/m2 D1, Cape 625 mg/m^2^ D1-21, q 3 wk), patients are randomised 1:1 to OXCAP-RT (oxaliplatin 85 mg/m^2^ Day 1,15,29; capecitabine 625 mg/m^2^ twice daily on days of RT; RT-45 Gy/25 fractions/5 weeks) or CarPac-RT (Carboplatin AUC2 and paclitaxel 50 mg/m2 Day 1,8,15,22,29; RT-45 Gy/25 fractions/5 weeks). Restaging CT/PET-CT is performed 4–6 weeks after CRT, and a two-phase oesophagectomy with two-field lymphadenectomy is performed six to eight weeks after CRT. The primary end-point is pathological complete response rate (pCR) at resection and will include central review. Secondary endpoints include: recruitment rate, toxicity, 30-day surgical morbidity/mortality, resection margin positivity rate and overall survival (median, 3- and 5-yr OS. 76 patients (38/arm) gives 90% power and one-sided type 1 error of 10% if patients on one novel treatment have a response rate of 35% while the second treatment has a response rate of 15%. A detailed RT Quality Assurance (RTQA) programme includes a detailed RT protocol and guidance document, pre-accrual RT workshop, outlining exercise, and central evaluation of contouring and planning. This trial has been funded by Cancer Research UK (C44694/A14614), sponsored by Velindre NHS Trust and conducted through the Wales Cancer Trials Unit at Cardiff University on behalf of the NCRI Upper GI CSG.

**Discussion:**

Following encouraging results from previous trials, there is an interest in neo-adjuvant chemotherapy and CRT containing regimens for treatment of oesophageal adenocarcinoma. NEOSCOPE will first establish the efficacy, safety and feasibility of two different neo-adjuvant CRT regimens prior to a potential phase III trial.

**Trial registration:**

Eudract No: 2012-000640-10. ClinicalTrials.gov: NCT01843829.

## Background

In the UK, about 7500 patients are diagnosed with oesophageal cancer each year, of which less than a quarter have resectable disease at diagnosis [1]. The most common treatment strategy in the UK over the past 10 years has been neo-adjuvant chemotherapy in the form of 2 cycles of cisplatin and 5-fluorouracil [5FU] prior to surgery. This is based upon the results of the MRC OE02 trial, which demonstrated a 6% survival benefit at 5 years for this approach over surgery alone. The data suggests this improvement was driven by improved local control rather than reduced metastatic spread. However, the overall survival is still poor, just 23% of patients surviving 5 years from treatment [2]. The MRC OE05 trial compared 4 cycles of neo-adjuvant ECX [epirubicin, cisplatin, capecitabine] chemotherapy to two cycles of cisplatin/5FU. It recruited over 800 patients and the final results are awaited.

Oesophageal cancer predominantly presents with locally advanced or metastatic disease. Even those found to be suitable for surgery usually present with stage III disease (at least T3 with lymph node metastases). The oesophagus lacks a serosal surface and tumours frequently threaten the circumferential resection margin (CRM). Disease present at or within 1 mm of the circumferential resection margin (CRM) (R1 resection) occurs in more than 50% of stage III cases treated by surgery alone [3,4] and is a poor prognostic factor. In the OE02 study, the 3-year and the median survival for patients with R0 and R1 resection were reported as 42.4% *vs.* 18% and 2.1 years *vs*. 1.1 years, respectively [2]. Like in rectal cancer, neo-adjuvant chemoradiotherapy [CRT] has the potential to downstage tumours that threaten CRM and potentially improve cure rates.

The concept of neo-adjuvant CRT had been previously rejected in the UK based on the increased mortality reported in previously conducted trials of pre-operative CRT, and the fact that all but one trial had failed to show a benefit in overall survival [5]. The landscape of neo-adjuvant CRT has recently changed with the reporting of the CROSS trial [6] which showed no increase in peri-operative mortality and a near-doubling of median survival compared to surgery alone. The data for benefit of pre-operative CRT for squamous cell carcinoma was so compelling that this is now standard of care. The CROSS trial has regenerated enthusiasm for neoadjuvant CRT trials in the UK and a national oesophageal workshop held in 2012 identified neo-adjuvant CRT as a key area of future research within the UK [7]. This has led to the development of the NEOSCOPE trial, which is evaluating CRT as an addition to pre-operative chemotherapy in adenocarcinoma of the oesophagus.

### Rationale

Previous trials of pre-operative CRT typically involved cisplatin-5FU based regimens and reported mortality rates of around 10% [8]. The CROSS trial, a randomised phase III study, compared surgery (S) alone to neo-adjuvant CRT (CRT-S) in 368 patients with operable oesophageal or gastro-oesophageal junction tumours (of whom approximately three quarters had adenocarcinoma and one quarter squamous cell carcinoma histology). The CRT regimen consisted of weekly carboplatin [AUC 2] and paclitaxel [50 mg/m2] concurrent with radiotherapy [41.4 Gy in 23 fractions]. This trial has shown a superior OS in favour of the CRT-S arm [OS 49 *vs.* 24 months, Hazard Ratio (HR) 0.657, p = 0.003], a pathological complete response [pCR] rate of 29%, with no increase in surgical mortality (4% in both groups) [6]. The R0 resection rates in the S and CRT-S arms were 69% and 92%, respectively [p < 0.001]. Of the 178 patients assigned to the CRT-S arm, 162 (91%) completed protocol treatment of five cycles of chemotherapy and 164 (92%) received full dose radiotherapy. The study reported a low incidence of Grade 3/4 CRT toxicity [haematological 7%; non-haematological 13%]. Only one death was reported in the CRT arm, probably due to perforation of the oesophagus accompanied by major haemorrhage in the absence of thrombocytopenia. The results of this study, performed in patients with a similar stage and tumour morphology to those in the UK, would suggest that where neo-adjuvant CRT is delivered safely, this leads to a significant improvement in outcome. Weekly carboplatin/paclitaxel/RT is a novel regimen, but although promising in its outcome, it had not been compared against the current standard of care for this disease, i.e. a neo-adjuvant platinum-5FU based chemotherapy or CRT.

Oxaliplatin has now been shown to be at least equivalent to cisplatin in advanced upper GI cancers, can be given as a convenient 2 hour infusion and has a more favourable toxicity profile compared to cisplatin [9]. Oxaliplatin based CRT has been tested in Phase II trials, both in the neo-adjuvant and definitive treatment of oesophageal cancer [10-16]. The PRODIGE5/ACCORD17 trial included patients selected to receive definitive CRT for oesophageal cancer [17]. In this multi-centre phase 2/3 trial, 267 patients were randomised to either FOLFOX (n = 134) or cisplatin-5FU (n = 133) in combination with RT. The median progression-free survival was comparable (FOLFOX group 9.7 months, Cisplatin-5FU group 9.4 months, p = 0.64) with no significant differences in rates of Grade 3/4 toxicity and lower number of toxic deaths associated with the FOLFOX arm (1 vs 6, p = 0.06). The authors concluded that FOLFOX-based CRT might be a more convenient option for patients with localised oesophageal cancer. In summary, studies of oxaliplatin–based CRT show promising activity with acceptable toxicity, may be potentially safer than cisplatin-based regimen, and therefore justify formal testing in a pre-operative study.

### Study objectives

NEOSCOPE is a randomised phase II study which will test the safety (with regard to post-operative morbidity/mortality), efficacy (determined by pathological complete response in the resected specimen, pCR) and feasibility of recruiting to a randomised multi-centre trial of neo-adjuvant CRT in the UK.

The randomised phase II design allows us to test two differing radiosensitiser schedules [carboplatin/paclitaxel and oxaliplatin/capecitabine] with non-overlapping toxicities. The study is aimed to identify a safe and effective regimen that can be taken forward to a future Phase III trial where neo-adjuvant CRT will be compared with neo-adjuvant chemotherapy in patients with locally advanced oesophageal cancer at high risk of R1 disease at surgery.

Additionally, blood and tissue samples will be collected for translational research and exploratory sub-studies will investigate whether PET/CT improves the accuracy and reproducibility of target volume delineation and the role of Image Guided Radiotherapy (IGRT) in improving the therapeutic index in oesophageal cancer.

## Methods/Design

### Study design

**Figure 1:**
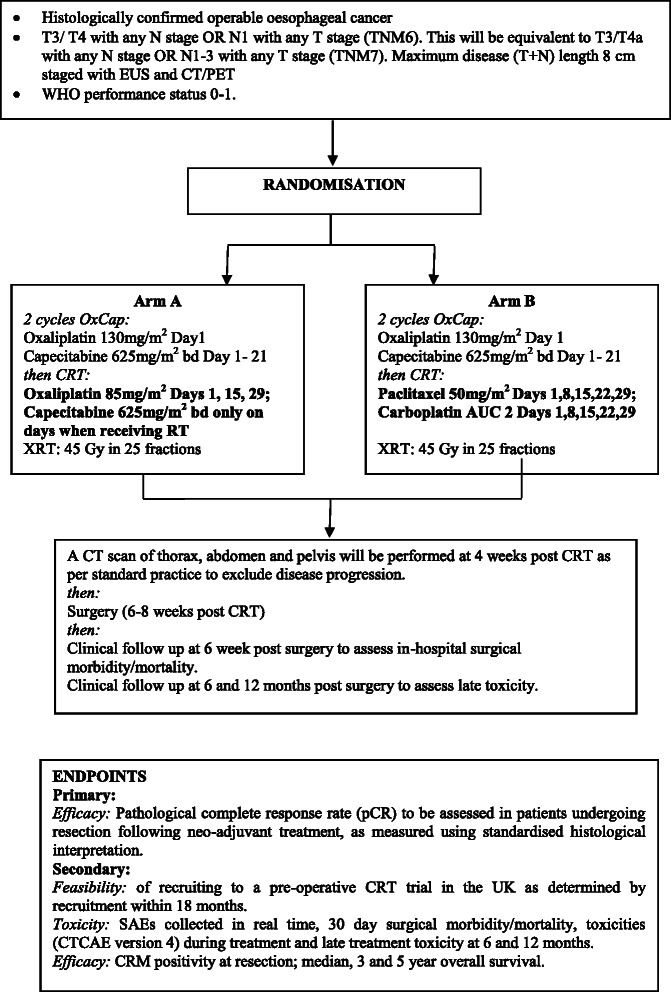
**Trial schema.**

NEOSCOPE is a two arm, open, randomised Phase II trial. It is being run in approximately 19 participating centres across the UK. Eligible patients will have histologically confirmed adenocarcinoma of the oesophagus and have been chosen to receive neo-adjuvant CRT by an accredited multidisciplinary team (MDT) including a specialist Upper GI surgeon (see Table 1 for detailed inclusion/exclusion). At randomisation, participants are assigned to either the OXCAP-RT or CarPac-RT using a 1:1 allocation ratio (see Figure 1). Both arms receive induction OXCAP chemotherapy consisting of 2 cycles of oxaliplatin 130 mg/m2 Day 1, Capecitabine 625 mg/m2 bd Day 1–21, q 3wk). Subsequent to induction chemotherapy, all patients will receive radiotherapy 45 Gy delivered in 25 daily fractions on weekdays only, by use of conformal radiotherapy planning. Patients allocated to the OXCAP-RT arm will receive concomitant oxaliplatin 85 mg/m2 by intravenous infusion on day 1,15,29 and capecitabine 625 mg/m2 twice daily on days of radiotherapy; patients on the CarPac-RT arm will receive Carboplatin AUC2 and paclitaxel 50 mg/m2 by intravenous infusion on days 1,8,15,22,29.

**Table 1:** **Inclusion and exclusion criteria for the NEOSCOPE trial**

***Inclusion criteria***	
	Patients meeting any of the following criteria may be included in the trial:
1.	Histologically confirmed operable oesophageal cancer [adenocarcinoma]
2.	T3/T4 with any N stage OR N1 with any T stage (TNM6). This will be equivalent to T3/T4a with any N stage OR N1-3 with any T stage (TNM7). T4a tumours should;
	a. involve only the diaphragm or crura, or
	b. invade only the mediastinal pleura, or
	c. breach the gastric serosa (TNM 7).
	Tumours with nodal disease (N1-3) affecting the origin of the left gastric and splenic artery with the coeliac axis (formerly staged as M1a in TNM 6) can be included.
3.	Maximum disease (T + N) length 8 cm staged with EUS and CT/PET with maximum extent of primary disease below the gastro-oesophageal junction being 3 cm.
4.	WHO performance status 0–1 and patient fit to be treated with combined modality therapy (chemotherapy and radiotherapy prior to surgery).
5.	Adequate respiratory and cardiac function: FEV1 > 1.5 litres and cardiac ejection fraction ≥50% on echocardiography or MUGA. These assessments should normally be performed within 4 weeks prior to randomisation. CPEX testing is allowable but must not replace the above investigations. Patients who have had their assessments done over 4 weeks prior to randomisation or have had borderline results may still be eligible provided that they have approval from the CI through the NeoSCOPE trial team.
6.	Adequate haematological, renal, and hepatic function:
	a. Liver function tests ≤1.5 × ULN
	b. White blood cell count ≥ 3 × 10^9^/l; platelets ≥ 100 × 10^9^/l.
	c. Glomerular filtration rate (GFR) >50 ml/minute calculated or measured.
	The above assessments should normally be performed within 1 week prior to randomisation. Patients who have had their assessments done over 1 week prior to randomisation or have had borderline results may still be eligible provided that they have approval from the CI through the trial team.
7.	The patient has provided written informed consent.
8.	The patient is at least 18 years old.
***Exclusion criteria***	
	If any of the following criteria apply, patients cannot be included in the trial:
1.	Oesophageal cancer with histology other than adenocarcinoma
2.	Uncontrolled angina pectoris, myocardial infarction within 6 months, heart failure, clinically significant uncontrolled cardiac arrhythmias, or any patient with a clinically significant abnormal ECG.
3.	Patients with any previous treatment for oesophageal carcinoma.
4.	Siewert type 3 oesophago-gastric tumours.
5.	Lower limit of the endoscopically visible primary tumour should not involve stomach for more than 3 cm distal to the gastro-oesophageal junction.
6.	T4 tumours invading contiguous structures other than diaphragm, crura or mediastinal pleura.
7.	Patients with disease in any of the following areas on the CT scan, EUS or other staging investigation:
	a. Evidence of metastases in liver, lung, bone or other distant metastases.
	b. Abdominal para aortic lymphadenopathy >1 cm diameter on CT or >6 mm diameter on EUS.
	c. Invasion of tracheo-bronchial tree, aorta, pericardium or lung.
8.	Lymphadenopathy encasing the coeliac axis (as described above, patients with single nodes lying anterior to the origin of the splenic artery and anterior to the origin of the coeliac axis are not excluded).
9.	Any patient with a single significant medical condition which is thought likely to compromise his or her ability to tolerate any of the above therapies.
10.	Specific contra-indications to surgery, chemotherapeutic agents (including known allergies to chemotherapy) or radiotherapy.
11.	Patients with another previous or current malignant disease which in the judgement of the treating investigator is likely to interfere with treatment or the assessment of response.
12.	Pregnant or lactating women and fertile women who will not be using contraception during the trial.

### Participant eligibility

Eligible patients will have been diagnosed with adenocarcinoma of the oesophagus, or Siewert Type 1 or 2 tumour of the gastro-oesophageal junction (GOJ) and selected for treatment with neo-adjuvant CRT by an appropriate specialised MDT. Such patients may enter the trial if they meet all inclusion and none of the exclusion entry criteria (see Table 1).

Staging investigations should include multi-slice/spiral CT scan of thorax and abdomen (neck and pelvis optional) with IV and oral contrast/water, maximum slice width 5 mm, endoscopic ultrasound (EUS), PET/CT scan (recommended but not essential) and laparoscopy (where clinically indicated – usually for lower third and GOJ tumours). All staging investigations should be ideally completed within four weeks prior to randomisation. However if this is not possible, the last staging investigation, which may include CT, EUS, PET/CT, or laparoscopy, should normally be performed within four weeks prior to randomisation. Cardiac and respiratory assessment (FEV1 using spirometer, cardiac ejection fraction using echocardiogram or MUGA and ECG) should also be performed within four weeks prior to randomisation. Within one week prior to randomisation the following screening assessments should be performed: a history and physical examination (to include height, weight and WHO performance status); full blood count; serum renal, liver and bone profile (including serum magnesium); glomerular filtration rate; electrocardiogram; and pregnancy test in females of child bearing age.

### Sample size considerations

The sample size calculations are based on the maximum of two binomial random variables and follow the ideas of Dunnett [18]. The primary outcome of the study is rate of pathological complete response in the resection specimen. A response rate of 15% would not be sufficiently large to warrant further investigation, while a response rate of 35% is considered worthwhile. Patients will be randomised 1:1 to each of the two treatment arms and the total sample size of the study is 76 (38 patients/arm). This design is based on a one-sided type I error of 10% and a power of 90% of achieving significance if patients on one novel treatment have a response rate of 35% while the second treatment has a response rate of 15%. This specification ensures that a power greater 90% is achieved if both treatments have a worthwhile effect of 35%. The study will seek to recruit a maximum total of 85 patients to account for a 10% drop-out rate before resection.

### Method of randomisation

Wales Cancer Trials Unit will randomise patients centrally using the method of minimisation with a random element. This will ensure balanced treatment allocation by a number of clinically important stratification factors. Randomisation will have an allocation ratio of 1:1.

### Outcome measures

#### Primary outcome measure

Efficacy: Pathological complete response rate (pCR) to be assessed in the resected specimen following neo-adjuvant treatment using standardised work up of the resection specimen in the pathology department and standardised histological criteria for tumour regression grading including central pathology review of all specimens.

#### Secondary outcome measures

Feasibility: of recruiting oesophageal cancer patients to a pre-operative CRT trial in the UK as determined by recruitment within 18 months.

Toxicity: SAEs collected in real time, 30 day surgical morbidity/mortality, toxicities (CTCAE version 4.03) during treatment and late treatment toxicity at 6 and 12 months.

Efficacy: CRM positivity (as defined by the Royal College of Pathologist’s guidelines), resection rate and overall survival (median, 3 and 5 year).

### Data collection

Participants will be seen at hospital at randomisation, the end of each treatment cycle, 4 weeks post CRT, 30 days post-surgery and then at month 6 and 12 post- surgery. Research staff at the hospitals will be expected to complete trial CRFs which record evidence of primary and secondary outcome measures. Patients will be flagged with the Health and Social Care Information Centre for longer term follow up of survival. Data on quality of life and health economics is not being collected in this Phase II study.

### Statistical analysis

All analyses will be on an intention-to-treat basis, i.e. all patients randomised will be included, and all patients will be analysed according to their allocated group whatever treatment they received. Statistical analysis will include:

- Descriptive statistics of the patient characteristics within each treatment group
- A CONSORT flow diagram of enrolment, intervention allocation, and follow-up
- Tables of toxicities at each timepoint (baseline, end of CRT (including all those during treatment), 30 days post-surgery, and then during later follow up.
- Treatment compliance during each cycle (in terms of proportions of patients with delay/reduction to CRT) within each treatment group. An exploration of predictors of poor compliance will be performed.

When 10 patients have completed treatment, i.e. 5 patients in either arm, a safety review will be performed. Full SAE and toxicity by arm will be presented to the Independent Data Monitoring Committee (IDMC) for a recommendation as to whether or not to continue recruitment. Additionally, upon any event of 30-day postoperative mortality, the IDMC and Trial Management Group (TMG) will be notified to discuss whether or not the trial should continue. The TMG will consider making a change to the radiotherapy protocol if necessary.

A full statistical analysis plan will be defined before the first interim analysis of the trial. Upon completion of recruitment and follow up, the pCR rates and post- operative mortality rates will be calculated by arm. The following rules will be used to decide whether or not there is sufficient evidence to warrant a future Phase III trial:

- If fewer than 10 patients achieve a pCR to either treatment, no treatment is taken forward to a Phase III trial.
- If 10 or more patients achieve a pCR to treatment A but fewer than 10 patients achieve a pCR to treatment B, treatment A is taken forward to a Phase III trial.
- If 10 or more patients achieve a pCR to treatment B, but fewer than 10 patients achieve a pCR to treatment A, treatment B is taken forward to a Phase III trial.
- If both treatments have 10 or more patients achieve a pCR, the treatment with higher response rate is taken forward to a Phase III study provided the post operative mortality is less than 5% in both arms. If post operative mortality is > 5% for one of the treatments while the mortality is below 5% for the other, the treatment with the lower post-operative mortality is taken forward.
- If both arms show high pCR and similar mortality then toxicities will be used to help decide which arm to take forward to a future Phase III.

### Radiotherapy quality assurance

The aim of the RTQA programme is to ensure compliance with the protocol and consists of both pre-accrual and on-trial (individual case) components. A detailed radiotherapy protocol and quality assurance procedure (Radiotherapy Trials Quality Assurance (RTTQA)) has been incorporated into this trial in collaboration with the NCRI RTTQA group at Velindre Hospital. This can be accessed at http://www.rttrialsqa.org.uk/rttqa/?q = neoscope. Protocol development was an iterative process over several months with incorporation of comments from several upper GI oncologists. The definition of target volumes and margins for NEOSCOPE has been informed by three main sources: (i) the CROSS trial radiotherapy protocol (ii) European Organization for the Research and Treatment of Cancer (EORTC) recommendations for the use of naCRT in adenocarcinomas of the gastro-oesophageal junction and the stomach [19] and (iii) the radiotherapy protocol of Art Deco (A Randomised Trial of Dose Escalation in definitive chemoradiotherapy for patients with Oesophageal cancer), a definitive CRT trial run by the CROSS group (M. Hulshof, personal communication).

#### Pre-accrual RTTQA

Prior to trial participation centres were required to satisfactorily complete a pre-accrual benchmark outlining and planning case. Investigators were asked to outline a middle and lower 1/3 oesophageal case and to plan a pre-outlined lower 1/3 case, both according to the protocol. All relevant clinical information and diagnostic imaging were provided, facilitated by the UK RTTQA website (http://www.rttrialsqa.org.uk/). A consensus reference volume was used to assess conformity. All cases were reviewed by the RTTQA team and detailed feedback was provided, including screenshots of their volumes overlying the reference volume. Criteria were developed for pass, minor and major deviations. Centres were asked to resubmit failed cases until satisfactory. Planning case and RT process was also QAed and fed back on, in the same manner, with advice suggested for improvement where necessary.

#### Real time RTTQA

Individual case review is carried out prospectively for the 1^st^ case from each centre and for the first 20 cases, repeating the process if there was an issue, until there is a satisfactory submission. We have paid particular attention to areas of variation identified in the pre-accrual cases. We will feed back to centres within 3 working days. For all remaining cases we use ‘timely retrospective review’, where outlines and plans are reviewed within 2 weeks of the start of radiotherapy.

### Translational research

The clinical trial includes tissue sample collection for future translational research and the development/validation of biomarkers. Trial participants will be asked for additional optional consent to participate in this aspect of the study. The biobank for this clinical trial will consist of pre-treatment diagnostic biopsy tissue, including one or more formalin-fixed, paraffin-embedded blocks of tumour and one block of normal mucosa, and 2 × 10 ml EDTA blood samples to be taken within 14 days before the first day of cycle 1 of chemotherapy. All samples will be stored at the Wales Cancer Bank for future translational research.

#### Regulatory approval, sponsorship and current status

NEOSCOPE has been ethically approved by the Research Ethics Committee for Wales and has approval from the Medicines and Health Care Product Regulatory Agency to be conducted in the UK. The Wales Cancer Trials Unit, a Cancer Research UK core funded and UKCRC accredited Clinical Trials Unit, is coordinating the trial. Velindre NHS Trust is the sponsor for the trial. A Trial Steering Committee and an Independent Data Monitoring Committee has been set up to monitor the progress and safety of the study. The NEOSCOPE Trial Management Group, including clinicians, clinical trial unit staff, patient representatives, nursing and pharmacy representatives, carry out the day-to-day running of the trial. The full trial protocol can be accessed at http://www.rttrialsqa.org.uk/rttqa/?q=neoscope.

NEOSCOPE is registered with Eudract No: 2012-000640-10 and Clinical trial information: NCT01843829.

The study opened to recruitment in September 2013, is open in 19 centres across UK and as of 8 September 2014 had recruited 43 patients.

## Discussion

This Cancer Research UK funded trial aims to define a safe and effective pre-operative CRT regimen in oesophageal adenocarcinoma to take forward to a phase III trial. The OE05 study, a randomised phase III trial, which recruited over 800 patients and compared 2 cycles of cisplatin-5FU chemotherapy to 4 cycles of ECX (epirubicin, capecitabine, cisplatin), is due to report later this year and is expected to define the optimal neo-adjuvant chemotherapy regimen. It is proposed that the best arm of NEOSCOPE will be taken forward to a phase III trial against the best arm of OE05. This study also builds on the high quality RTTQA programme which was built around the SCOPE trial [20] and aims to standardise the delivery of pre-operative radiotherapy across the UK.

The use of neo-adjuvant CRT in oesophageal cancer has been tested in a number of studies, which have been heterogeneous in design, size and treatment regimen tested. Nevertheless, a meta-analysis of randomised trials has shown that this approach increases R0 resection rates, reduces loco-regional recurrence and improves survival compared with surgery alone [8]. There has been only one randomised phase III trial comparing pre-operative chemotherapy with pre-operative CRT. This study by Stahl *et al.* aimed to recruit 354 patients to detect a 10% improvement in 3-year overall survival [OS] in favour of CRT [from 25% to 35%] but closed early due to poor recruitment (126 patients recruited over 5 years). Nonetheless, it showed a non-significant trend towards improved 3-year survival in favour of CRT [47.4% v 27.7%, p = 0.07] [21].

Through better selection of patients, improved peri-operative care and centralisation of upper gastro-intestinal surgical services, there has been a significant reduction in post-operative mortality. In the MRC OE02 trial, the post-operative mortality was 10% in patients receiving either neo-adjuvant chemotherapy or surgery alone [22]. In the 3rd Annual Report of the UK National Oesophago-Gastric Cancer Audit (26th March 2012), the post-operative mortality was 3.8% and many large surgical centres now have rates of in-hospital mortality of < 3% [23]. Neo-adjuvant CRT has been historically associated with higher post-operative mortality. In the Urschel meta-analysis, there was a non-significant increase in peri-operative mortality [1.72 (0.96, 3.07; p = 0.07)] and increase in all-treatment mortality that was of borderline significance [1.63 (0.99, 2.68; p = 0.053)] [8]. In the Stahl trial of neo-adjuvant CRT *vs*. neo-adjuvant CT, there was a trend towards increased post-operative mortality [5 of 49 (10.2%) *vs*. 2 of 52 (3.8%, p = 0.26] [21]. Although only recently reported, this study was designed in the 1990s and opened to recruitment in November 2000. Three-dimensional conformal radiotherapy was recommended but not mandated as part of this trial. This, together with the lack of pre/on-trial RT quality assurance, may have contributed to increased post-operative morbidity and mortality. Contrary to this, the CROSS trial found no significant increase in peri-operative mortality, which probably reflects the use of patient selection and improvement in surgical care and radiotherapy delivery over time.

In recent years, a great deal of work has been done to raise the standards of conformal radiotherapy for oesophageal cancer in the UK. Through a detailed protocol and quality assurance procedures, including test cases and on trial review of plans, the SCOPE 1 trial, a randomised trial of definitive CRT with cisplatin and capecitabine with/without Cetuximab, recruited greater than 250 patients from 44 radiotherapy centres and demonstrated that the UK can perform high quality, multi-centre RT based studies safely [24]. This trial has allowed UK investigators to gain significant experience in delivering quality assured CRT and this provides an ideal platform on which to build a neo-adjuvant study, and to investigate precise methods of tumour localisation such as CT/PET and image-guided radiotherapy [IGRT], all of which should allow safer and more accurate delivery of RT, reduction of radiation damage to organs at risk and ultimately reduce post-operative morbidity and mortality.

The current UK standard practice has been largely influenced by the results of the MRC OE02 trial and until recently, clinicians had been actively recruiting to the MRC OE05 trial. Concerns about increased post-operative mortality/morbidity from neo-adjuvant CRT as well as participation in the MRC OE05 trial had discouraged routine use of neo-adjuvant CRT. However, the MRC OE05 trial has now closed to recruitment, the outcome with neo-adjuvant chemotherapy alone remains poor and the CROSS trial demonstrated that neo-adjuvant CRT can be given with acceptable morbidity, and when done so, is associated with a significant survival advantage. The UK has now seen the development of a high quality upper GI RT Quality Assurance programme through the SCOPE 1 trial. This, along with better patient selection through new imaging techniques like PET-CT and EUS, and improvement in peri-operative care through centralisation of surgical services, is expected to lead to better outcome than reported in previous neo-adjuvant CRT trials.

Based on the above, there is a growing clinical consensus that the two strategies of neo-adjuvant chemotherapy and CRT containing regimens should be compared head-to-head in a prospective randomised controlled trial in oesophageal adenocarcinoma, particularly focussing on those patients who are at high risk of R1 surgical resection. NEOSCOPE will first establish the efficacy, safety and feasibility of two different neo-adjuvant CRT regimens.
